# Non-fibril form but not fibril form of human islet amyloid polypeptide 8–20 changes brain functions in mice

**DOI:** 10.1371/journal.pone.0296750

**Published:** 2024-01-05

**Authors:** Hinaho Suginoma, Ryuji Owada, Akiko Katano-Toki, Ayaka Mori, Jun Fujioka, Kazuhiro Nakamura

**Affiliations:** 1 Department of Laboratory Sciences, Gunma University Graduate School of Health Sciences, Maebashi, Gunma, Japan; 2 Department of Chemistry, Faculty of Science Division I, Tokyo University of Science, Shinjuku-ku, Tokyo, Japan; Instituto Mexicano del Seguro Social, MEXICO

## Abstract

Whether fibril formation increases or decreases cytotoxicity remains unclear. Aggregation of human islet amyloid polypeptide (hIAPP), a pivotal regulator of glucose homeostasis, impairs the function and viability of pancreatic β cells. Evidence suggests that low-order oligomers of hIAPP are more toxic to β cells than fibril. However, it remains unclear whether non-fibril form of hIAPP specifically alters brain functions. This study produced fibril and non-fibril forms from a single hIAPP 8–20 peptide. The non-fibril form-injected mice showed changes in spontaneous motor activities, preference for location in the open field and social behavior. In contrast, the fibril-injected mice showed no changes in these behavioral tests. In line with the behavioral changes, the non-fibril form led to impaired neurite outgrowth of cultured neuron-like cells and the loss of neurons in the mouse hippocampus. These findings suggest that non-fibril form but not fibril form of hIAPP changes brain functions.

## Introduction

Human islet amyloid polypeptide (hIAPP) is co-stored and co-secreted with insulin in the pancreas under physiological conditions [1] and, therefore, plays a physiological role in glucose homeostasis.

Although abundant IAPP is produced in pancreatic β cells, a lower amount of IAPP is produced in specific brain areas [2]. Additionally, peripheral IAPP crosses the blood-brain barrier [2]. Thus, IAPP is also found in the brain. Uptake [3] and binding experiments [4] using labeled IAPP-specified preferential brain regions. These regions include the area postrema, nucleus of the solitary tract, medial preoptic area, arcuate nucleus, ventromedial hypothalamus, dorsomedial hypothalamic nucleus, nucleus accumbens, and dorsal raphe. IAPP functions as a ligand for multiple subunits of the calcitonin receptor in the brain [5], and the calcitonin receptor is expressed in most brain regions [2]. Therefore, IAPP can potentially exert physiological effects on these brain regions. Indeed, IAPP in the brain has been suggested to affect eating and energy homeostasis [2]. Additionally, IAPP and calcitonin receptors in the medial preoptic area have been implicated in risk-taking maternal care [6] and affiliative social behaviors [7] in mice. Furthermore, IAPP contributes to the pain response in mice [8]. Even in humans, improvement in cognitive function has been associated with increasing quartiles of IAPP in plasma in a homebound elderly population [9].

In addition to the physiological role of IAPP, hIAPP can also exert toxicity in pancreatic β cells. Under chronic hyperglycemic conditions in type-2 diabetes, hIAPP can form amyloids on the cell membrane, which leads to dysfunction and death of pancreatic β cells [10]. hIAPP is also likely involved in brain pathology because a functional link between hIAPP and amyloid β (Aβ), a causative molecule of Alzheimer disease, has been proposed. Aβ inclusions are frequently found in pancreatic cells [11] and hIAPP deposition is also often observed in the hippocampus and locus coeruleus in Alzheimer disease patients with neuropathologic changes [11].

Regarding the relationship between the degree of aggregation of hIAPP before attachment to the cell surface and its toxicity to β cells, lag phase intermediates during the aggregation process of hIAPP that form low-order oligomers specifically lower the viability of β cells and upregulate pro-inflammatory cytokines and oxidative stress [12]. In the same study, hIAPP fibrils were less toxic [12]. However, whether the non-fibril but not the fibril leads to the brain dysfunction remains unclear.

In the present study, we prepared fibril and non-fibril forms from a single hIAPP 8–20 peptide.

The two hIAPP 8–20 preparations were injected into the mouse hippocampus and the mice were subjected to behavioral analyses. The results indicated that the non-fibril but not fibril form led to behavioral alterations.

## Materials and methods

### Peptide

The purity of the synthesized FITC-labeled hIAPP 8–20 (ATQRLANFLVHSS; GenScript Biotech Corporation, China) was 80%. The stock solution of the peptide in HFIP was prepared at 10 mg/ml. The stock solution was directly diluted with distilled water at 1 mg/ml (containing 10% HFIP and 90% water). Then, the diluted peptide was incubated at either 37 °C for 7 days or in room temperature (RT) for a couple of minutes. In an another preparation, the stock solution was dried using centrifugal evaporator. Then, distilled water was added to the dried peptide at 1 mg/ml and the peptide solution was incubated at 37 °C for 2 days.

### Transmission Electron Microscopy (TEM)

Five μl of peptide solution (1mg/ml) was blotted onto a carbon-coated Formvar 300 mesh copper grid for 2 min and was then negatively stained with saturated uranyl acetate. TEM images were taken using HT7800 Transmission Electron Microscope (Hitachi High-Tech Group, Tokyo, Japan) with a voltage of 80 kV.

### Dynamic light scattering (DLS)

DLS experiment was performed using peptides (1 mg/ml in water) in a sample cell in room temperature to determine the size of particles. Light scattering photometer, ELSZ2000 (Otsuka Electronics Co., Ltd., Osaka, Japan) was used for the experiment. A vertically polarized semiconductor laser operated at the wavelength of 660 nm was used as the incident beam, and the vertically polarized scattered light was detected at the scattering angle of 165.

### Cell culture

The subclone, PC12 HS purchased from RIKEN BRC is highly sensitive to NGF. The cell line was cultured at 37 °C with 5% CO2. 1 x 10^4^ PC12 cells were plated in 24 well plates. One day after adding aggregates to the culture medium to a final concentration of 10 μg/ml, the cells were cultured in Dulbecco’s Modified Eagle Medium (11965118; Thermo Fisher Scientific, Waltham, MA) containing 1% fetal bovine serum (FBS)(04-111-1A, Biological Industries Ltd., Kibbutz Beit-Haemek, Israel), 0.25% BSA (012–23881, FUJIFILM Wako Pure Chemical Corporation, Tokyo, Japan), 1% penicillin-streptomycin mixed solution (168–23191, FUJIFILM Wako Pure Chemical Corporation, Tokyo, Japan) and NGF (SRP3015, Sigma-Aldrich, Inc., St. Louis, MO)(50 ng/ml) for 5 days to induce differentiation.

Visible and fluorescent images were captured using a BZ-9000 microscope (Keyence, Osaka, Japan) and the morphology of the cells was quantitatively analyzed as previously described [13]. The original images were acquired by Image J software (National Institutes of Health, Bethesda, MD, United States) and the actual scale in the original images was reflected in the acquired images. Neurites of PC12 cells were traced using tools of the software. Because the length of less than 1 μm was difficult to trace, the neurites longer than 1 μm were measured. Then, the total length of neurites per cell was calculated. The experimenter who cultured PC12 cells was different from those who analyzed the data.

### Mice

The experimental protocol was in accordance with the relevant guidelines and regulations of the NIH and approved by the Animal Resource Committees of Gunma University (Approval number, 22–061). The number of mice used for the experiments was a bare minimum to obtain reliable data. The mice were maintained under specific pathogen-free conditions. The room temperature was maintained at 23°C, and the light/dark cycle was constantly maintained at 12h each. Four to five mice were housed per cage.

For peptide injection, male and female ICR mice at 2 months of age (Japan SLC, Inc., Hamamatsu, Japan) were randomly divided into groups and were anesthetized with isoflurane. After fixing the head with a stereotaxic instrument, 2 μL of the peptide (100 μg/ml in PBS) was injected into the right hippocampus (bregma: AP, -2.00 mm; ML, +1.3 mm; DV, -2.2 mm), according to the instructions provided in a previous study [14]. Behavioral tests were performed the following day and 1 month later. We made every effort to minimize the suffering in mice. For instance, a short operation time (less than 10 min) and a small incision area of the skin (smaller than 1 cm) were applied for injection. In addition, cervical dislocation was applied for euthanasia after the experiments.

### Imaging

The brains were fixed with 4% paraformaldehyde in PBS (T900, Takara Bio Inc., Kusatsu, Japan). After dehydration with 30% sucrose in PBS, coronal sections of the rostral hippocampus 25 μm in thickness were prepared using a cryostat. For Nissl staining, the sections were stained with 0.1% cresyl violet solution (41022, MUTO PURE CHEMICAL CO., LTD., Tokyo, Japan) for 20 min at 60°C. Visible images were captured using a BZ-9000 microscope. Sections with the same rostral-caudal levels between groups were subjected to quantification of the thickness of the pyramidal cell layer in the CA3c region, close to the lateral end of the granule cell layer in the dentate gyrus [15]. The Image J software was used for quantification.

### Behavioral tests

The open-field test for 10 min was performed as described previously [16]. The size of the open-field apparatus (O’HARA & Co., Ltd., Tokyo, Japan) was 50 cm × 50 cm × 50 cm. The system automatically measured spontaneous locomotion in the mice. The floor of the open field was covered with black paper to detect the movement of the white mouse. Before starting the test, mice were placed in the central region of the open field. Subsequently, the test was initiated. The parameters measured in the test included total walking distance, duration of movement during the test session, total number of movements, speed during movement, walking distance per movement, duration per movement and the times mice stayed in the wall-side and center area.

The three-chambered social approach task was performed as previously described [17]. The apparatus (20 × 40 × 23 cm; O’HARA & CO., LTD., Tokyo, Japan) consisted of equal-sized left, center, and right chambers. The mice could freely enter the next chamber. A small wire cage was placed in the left and right chambers. In the first session, the subject mouse was placed in the center chamber, and the time spent around the empty wire cage in the left chamber (left cage) was recorded for 5 min. Before the second session, a different mouse was placed in a wire cage in the left chamber. Subsequently, the subject mouse was immediately placed in the center chamber, and the time the subject mouse spent around the left wire cage was recorded for 5 min as an index of social approach. In the third session, a novel, the unfamiliar mouse was placed in the wire cage in the right chamber, leaving an old familiar mouse in the left wire cage. The time the mouse spent around the left wire cage was recorded for 5 min as an index of social memory.

The protocol and apparatus of the elevated plus maze test were the same as those described previously [13]. Entering into the arms was defined as when all four paws were on the arms. The total time spent in the open arms was measured for 10 min.

The apparatus for the light-dark box test was ImageJ LD1 (O’HARA & CO., LTD. Tokyo, Japan). The size of the boxes was 200 mm × 200 mm × 250 mm. A small hole (30 × 50 mm) enabled the subject to visit from one room to another. The mice were initially placed in a dark box, and the time taken to first enter the light box was measured.

The same mice were used to conduct behavioral tests one day and one month after the injection. The tests were performed in the following order: three-chamber test, open field test the day after the injection, elevated plus maze test, light-dark box test, three-chamber test and open-field test one month after the injection.

### Statistical analysis

The values expressed are the mean in the graphs and the error bars represent the standard deviation (SD). Normality was assessed by Kolmogorov-Smirnov and Shapiro-Wilk tests. When normality was met in either one of the two tests, statistical significance was studied using one-way ANOVA or two-way ANOVA followed by Scheffe posthoc test. When normality was not met in both tests, the Mann-Whitney U test or Friedman test followed by the Scheffe test was used. Statistical significance was set at p < 0.05.

## Results

### Production of hIAPP 8–20 peptides with different degrees of aggregation

Although hIAPP is prone to aggregation, rodent IAPP may not necessarily aggregate [18]. Therefore, we used the hIAPP. The full-length form of hIAPP is a peptide having 37 amino acids [19]. Because a previous study reported that even hIAPP 8–20 forms cross-β nanofibrils [20], we decided to use hIAPP 8–20. To facilitate peptide detection, FITC was conjugated into the peptide. The degree of aggregation of the peptides depends on their concentration. Since our previous studies using multiple peptides verified that a concentration of 1 mg/ml in water was adequate to form aggregates [13, 16], we used this concentration and tried to prepare both fibril and non-fibril forms from a single hIAPP 8–20 peptide.

**Fig 1:**
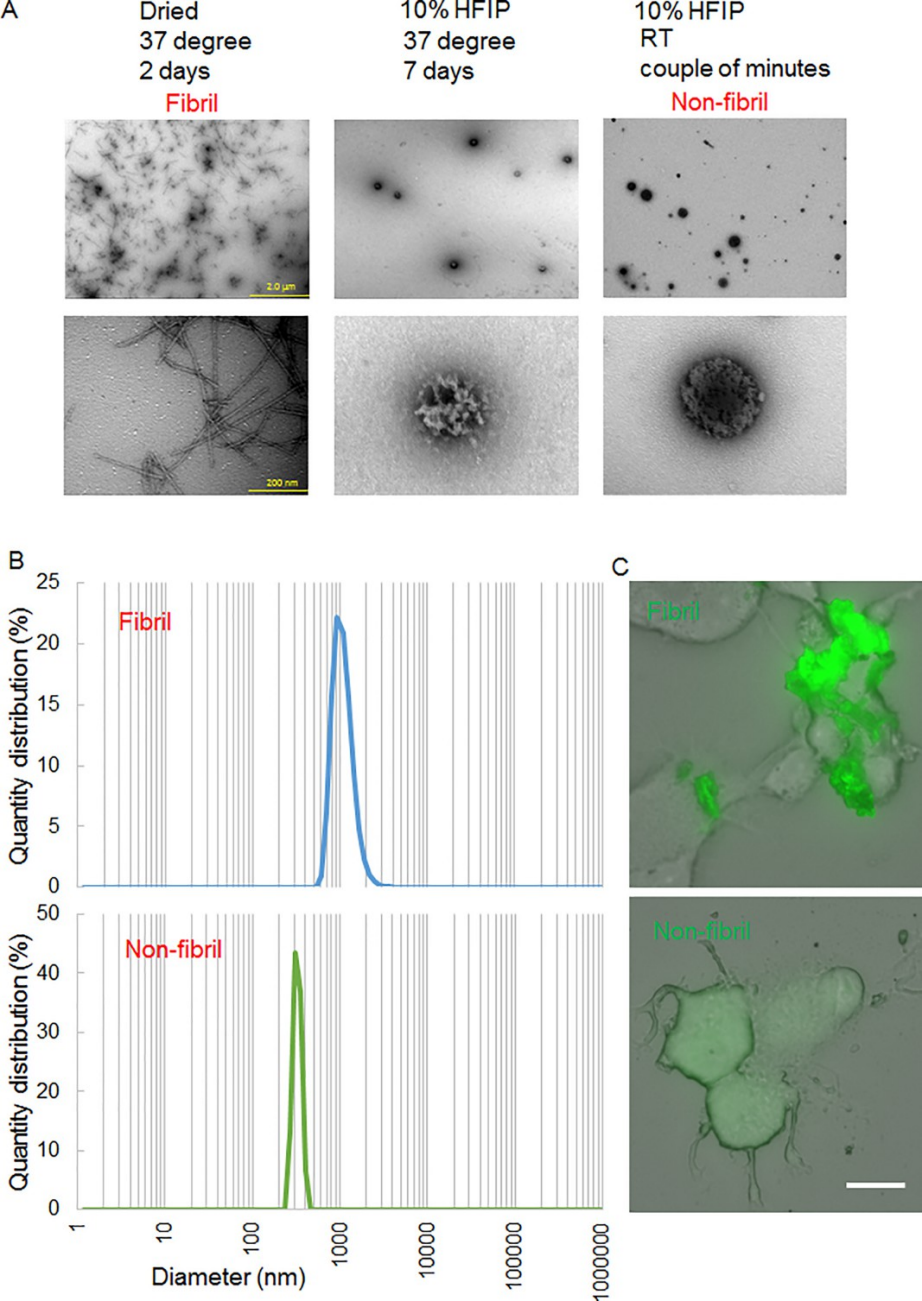
The appearance of hIAPP 8–20. (A) Low (Top) and high (Bottom) magnifications of TEM images. Stock solution of the peptide was directly diluted with distilled water at 1 mg/ml (including 10% HFIP). Then, the diluted peptide was incubated at either 37°C for 7 days or in RT for a couple of minutes (non-fibril form). In another preparation, a stock solution of the peptide was dried using a centrifugal evaporator. Then, distilled water was added to the dried peptide at 1 mg/ml and the peptide solution was incubated at 37°C for 2 days (fibril). Scale bars, 2 μm (Top) and 200 nm (Bottom). (B) Quantity distribution of the fibril (Top) and non-fibril (Bottom) forms obtained from DLS data. (C) Fluorescence microscope images of the fibril (Top) and non-fibril (Bottom) forms of hIAPP 8–20 (green) applied to the culture media of PC12 cells. Fluorescent and visible images were merged. Scale bar, 20 μm.

The stock solution of the peptide in HFIP was prepared at 10 mg/ml. To produce the fibril, the stock solution was dried to evaporate HFIP, and distilled water was added to the dried peptide to 1 mg/ml. The peptide solution was incubated at 37 °C for 2 days. As shown in Fig 1A, a typical fibril structure was observed in TEM images. Thus, this protocol was applied to produce fibril.

Generally, short-time incubation of peptide solution in RT produces a non-fibril form. However, the non-fibril form in the absence of HFIP becomes fibril in a couple of days at 37 °C as evidenced above. In the following functional analyses, we apply the non-fibril form to culture medium and mouse brain where the temperatures are around 37°C. Therefore, we searched for a condition that does not induce fibril formation in 37°C. When the stock solution (100% HFIP) without drying is diluted with water to 1 mg/ml, the peptide solution contains 10% HFIP and 90% water. Then, the diluted peptide was incubated at either 37°C for 7 days or in RT for a couple of minutes. TEM images verified that even the peptide incubated at 37°C for 7 days did not produce fibril (Fig 1A). Instead, this condition resulted in the formation of a round-shape cluster (Fig 1A). This result indicates that HFIP interferes with fibril formation. Therefore, we incubated the peptide (1 mg/ml in 10% HFIP and 90% water) in RT for a couple of minutes to obtain a non-fibril form. TEM images of the non-fibril form showed a round-shape cluster (Fig 1A).

DLS measurement indicated that the round-shape cluster of the non-fibril form was 300 nm in diameter (Fig 1B). Size of the aggregates consisting of fibril was larger (1000 nm in diameter) than those of the non-fibril form (Fig 1B).

In the following functional analyses, the non-fibril form is applied to cultured cells and mouse brains, where the final concentrations of HFIP become lower than 10%. Therefore, the non-fibril form might be converted into fibril in the culture medium and the brain at 37°C. We then tested this possibility. The fibril and non-fibril forms were applied to the culture media of PC12 cells. The final concentrations of FITC-labeled hIAPP 8–20 and HFIP in the culture media were 10 μg/ml and 0.1%, respectively. The culture medium was replaced with fresh medium to exclude HFIP the next day and PC12 cells were kept at 37°C for 5 days more. As shown in Fig 1C, large aggregates consisting of FITC-labeled hIAPP 8–20 fibrils could be easily found around the cells. In contrast, faint FITC signals of non-fibril form were diffusely found on the cell surface. Thus, the appearances of the two forms of hIAPP 8–20 were entirely distinct, indicating that the non-fibril form seemed not to become fibril in culture media at 37°C.

Collectively, we had an opportunity to compare the effects of the fibril and non-fibril forms on cultured cells and mouse brains. In the following functional analyses, FITC was used for control of hIAPP 8–20. FITC was also first dissolved in HFIP. As the control for fibril, distilled water was added to the dried FITC so that it became the same molar concentration. To prepare the control for non-fibril form, FITC in HFIP was directly diluted with water.

### Injection of fibril into the hippocampus results in no changes in spontaneous motor activity, preference for location and social behavior the next day

**Fig 2:**
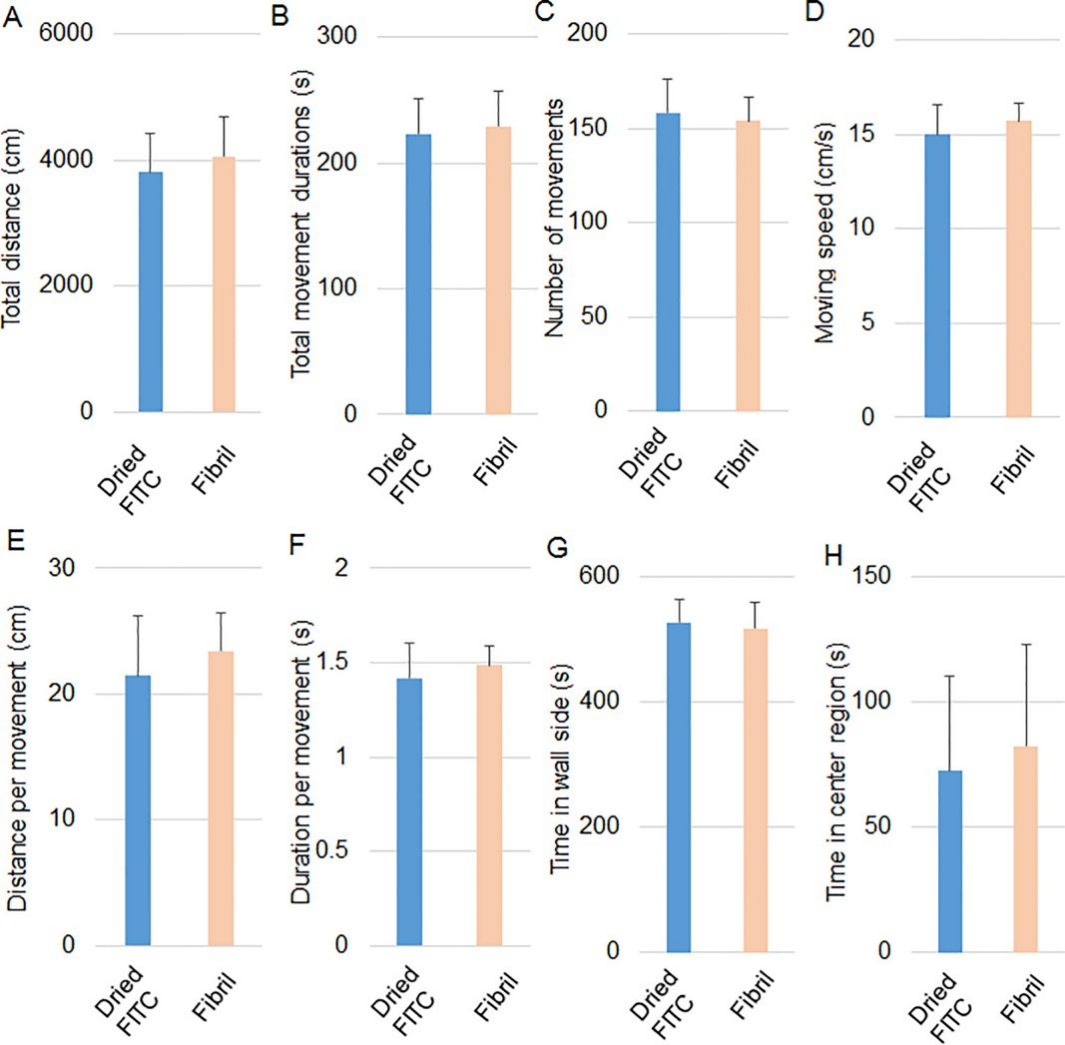
Performance of open field test of fibril-injected mice. The open field test was performed one day after the injection of FITC (without HFIP) and the fibril into the mouse hippocampus. The total walking distance (A), total moving durations (B), the total number of movements (C), moving speed (D), distance per movement (E), duration per movement (F), time mice stayed on the wall side (G) and time in the center area (H) in an open field were measured. n = 8 and 10 for mice with FITC and the fibril, respectively. Error bars represent SD. One-way ANOVA was applied for comparison between groups.

We screened the effects of the fibril and non-fibril forms on neurons using behavioral tests of mice the following day after the injection into the hippocampus. We first compared the behavioral results between fibril-injected mice and FITC (without HFIP)-injected mice. The temporal and spatial variabilities of gait patterns are associated with differences in the structure and function of the hippocampus and the primary sensorimotor cortex [21]. Thus, we assessed the pattern of spontaneous locomotion in the open field for 10 min. The parameters used were the total walking distance (Fig 2A), total moving durations (Fig 2B), the total number of movements (Fig 2C), moving speed (Fig 2D), distance per movement (Fig 2E) and duration per movement (Fig 2F) in an open field. There were no significant differences between the groups in all parameters, indicating no change in spontaneous locomotion in the open field by fibril.

Mice generally prefer the wall side area to the central area in the open field. Indeed, time spent in the wall side area was longer than in the center area in the two groups (Fig 2G and 2H). There were no differences in times in wall side and center areas between the groups. Thus, hIAPP 8–20 fibrils did not alter the preference for location in the open field.

**Fig 3:**
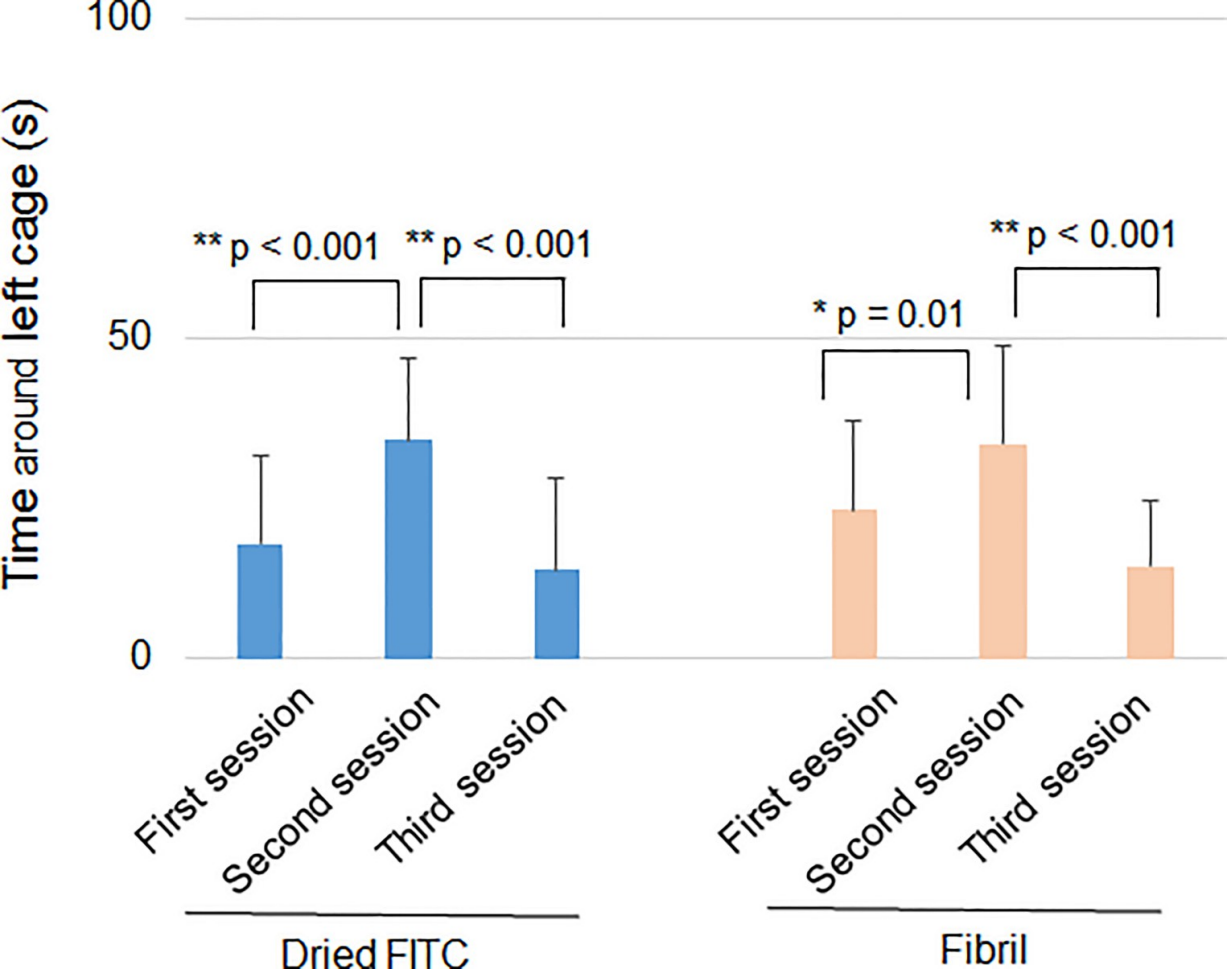
hIAPP 8–20 fibril in the hippocampus induce normal social behavior. The three-chamber test was done the following day after injection. The time spent around the left cage was measured during three sessions using mice given FITC (without HFIP) (n = 8) or hIAPP 8–20 fibril (n = 10) in the hippocampus. Two-way ANOVA followed by the Scheffe posthoc test was applied for comparison among the sessions. *p < 0.05; **p < 0.01.

The next test for the screening was a three-chamber test because the hippocampus is involved in social behavior and memory [22]. In the first session, the subject mouse freely explored the three chambers (left, center, and right) in the absence of different mice. This session can determine the preference of each chamber without the influence of the other mice. In the second session, the same test was performed for the subject mouse in the presence of a different mouse in a small wire cage in the left chamber. If the subject mouse showed a normal social approach, it tends to spend a long time around the left wire cage. Indeed, the FITC group significantly spent more time around the left wire cage in the second session than in the first session the next day after the injection (Fig 3). The difference was also significant in the fibril group (Fig 3), suggesting the normal social behavior of mice given fibril.

In the third session, a novel, the unfamiliar mouse was placed in a small wire cage in the right chamber, leaving an old familiar mouse in the left wire cage. If the social memory of the subject mouse is normal, the subject mouse no longer spends a long time in the left cage. Both FITC and fibril groups spent significantly less time around the left cage in the third session than in the second session (Fig 3). The result suggests that social memory was not impaired by fibril. Collectively, fibril did not induce behavioral changes the next day.

### Non-fibril form leads to changes in spontaneous motor activity, preference for location and social behavior

**Fig 4:**
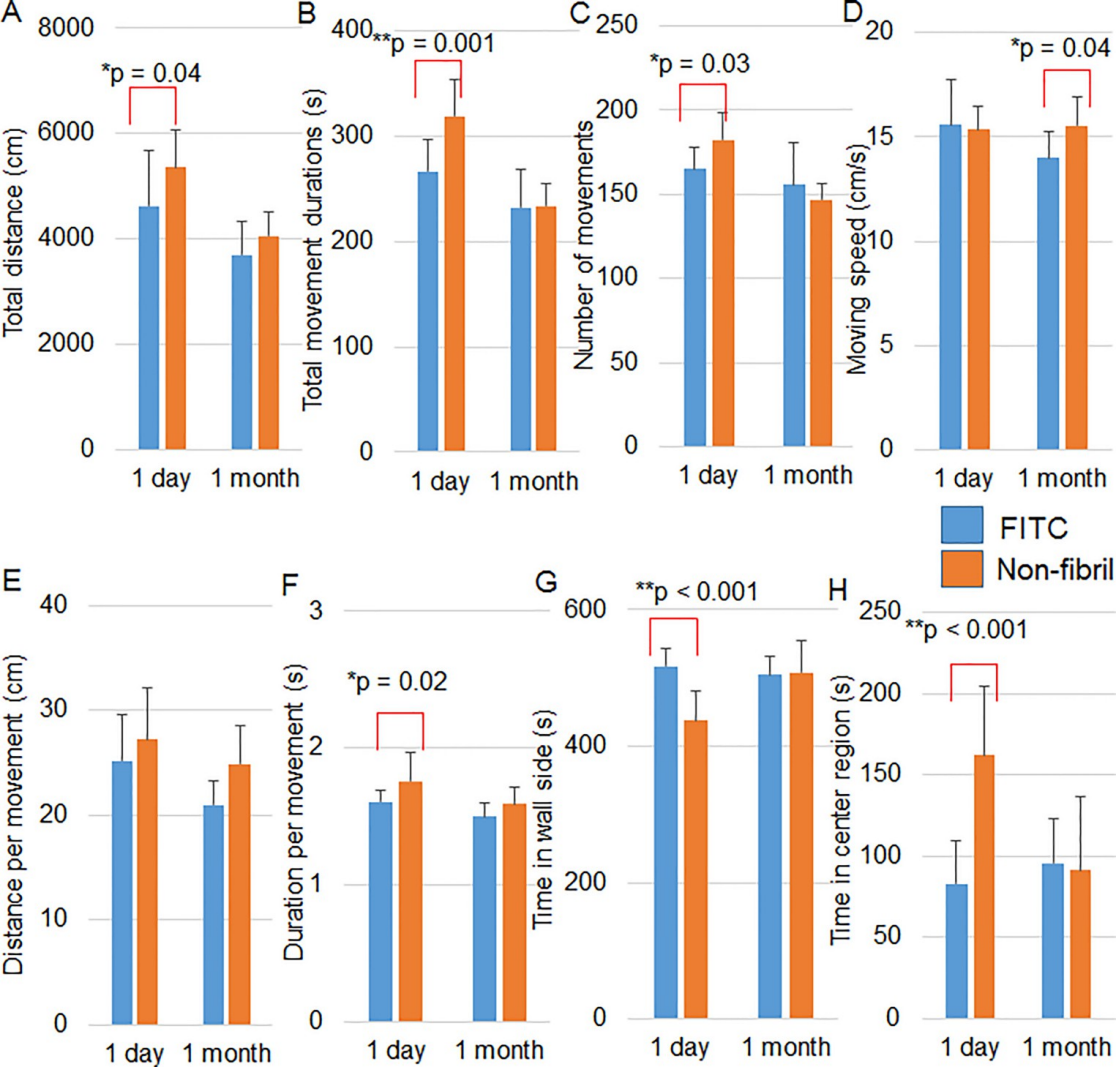
Injection of non-fibril form in the hippocampus leads to enhanced spontaneous motor activity and change in preference for location in an open field. The open field test was performed 1 day and 1 month after the injection of FITC (with HFIP) and the non-fibril form into the mouse hippocampus. The total walking distance (A), total moving durations (B), the total number of movements (C), moving speed (D), distance per movement (E), duration per movement (F), time mice stayed in the wall side (G) and time in the center area (H) in an open field were measured. n = 8 and 10 for mice with FITC and non-fibril form, respectively. Error bars represent SD. Two-way ANOVA (A-E. G, H) and Mann-Whitney U test (F) were applied for comparison between groups. *p < 0.05; **p < 0.01.

We then carried out the same behavioral tests the next day after injection with non-fibril form and compared the results with those of FITC (with HFIP)-injected mice. The total walking distance (Fig 4A), total movement duration (Fig 4B) and duration per movement (Fig 4F) were significantly longer and a number of movements (Fig 4C) was greater in non-fibril form-injected mice than in FITC-injected mice. In contrast, moving speed (Fig 4D) and distance per movement (Fig 4E) were comparable between the two groups. These results suggest enhanced activity of spontaneous locomotion of mice given non-fibril form the next day.

In addition, non-fibril form-injected mice spent a shorter time than FITC-injected mice on the wall side (Fig 4G). Conversely, the time in center areas was longer in non-fibril form-injected mice (Fig 4H). Thus, the non-fibril form altered the preference for location in the open field the next day.

Given the altered open-field performances one day after the injection, we examined whether the changes were also seen 1 month after the injection. As shown in Fig 4D, enhanced moving speed was recognized in mice given non-fibril form. However, there were no significant changes in other parameters. Thus, altered performances in the open field test in mice given non-fibril form were essentially transient.

**Fig 5:**
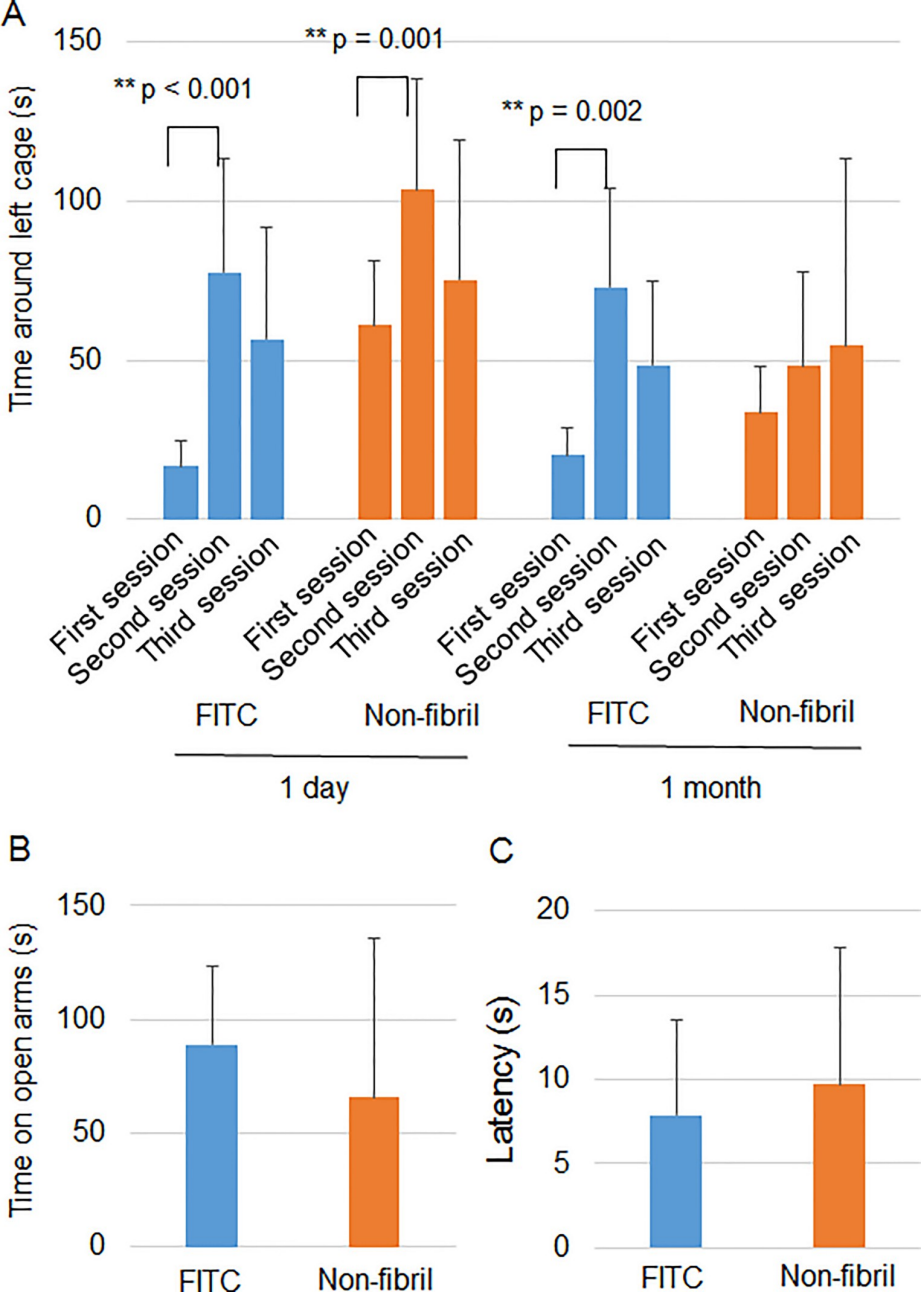
Non-fibril form in the hippocampus induces defective social behavior in mice. Behavioral tests were performed 1 day (A) and 1 month (A-C) after the injection of FITC (with HFIP) (n = 8) or the non-fibril form (n = 10) into the mouse hippocampus. (A) Three-chamber test. The time spent around the left cage was measured during three sessions. Two-way ANOVA followed by Scheffe analysis (1 day) or Friedman test followed by Scheffe analysis (1 month) was applied for comparison among the first, second and third sessions. (B) Time spent in the open arms in the elevated plus maze test. One-way ANOVA was applied for comparison between groups. (C) The latency in entering the light room was compared using the light-dark box test. One-way ANOVA was applied for statistical analysis. Error bars represent SD. **p < 0.01.

In the three-chamber test, the FITC group significantly spent more time around the left cage in the second session than in the first session both 1 day and 1 month after the injection (Fig 5A). The significant difference was also found in the non-fibril form group 1 day after the injection. However, the difference was not significant in the non-fibril form group 1 month after the injection (Fig 5A), suggesting defective social behavior of non-fibril form-injected mice 1 month later. The FITC group did not spend significantly less time around the left cage in the third session than in the second session both 1 day and 1 month after the injection (Fig 5A) and therefore, we could not assess the effect of non-fibril form on social memory.

Finally, we performed tests for anxiety using the same mice because the hippocampus has been suggested to mediate untrained anxiety reactions [23]. The tests were not performed the following day but one month after the injection because anxiety is likely to increase shortly after the operation. When mice are anxious, they spend a short time in the open arms in the elevated plus maze. The time spent in the open arms was comparable between the groups (Fig 5B). The second test was a light-dark box test. The mouse was first placed in a dark box, and the time taken to enter the light room was measured. No difference was observed between the groups (Fig 5C). Thus, no evidence of altered anxiety could be observed. Taken together, non-fibril but not fibril form of hIAPP 8–20 induced behavioral changes.

### Non-fibril form induces morphological abnormalities in vitro and in vivo

**Fig 6:**
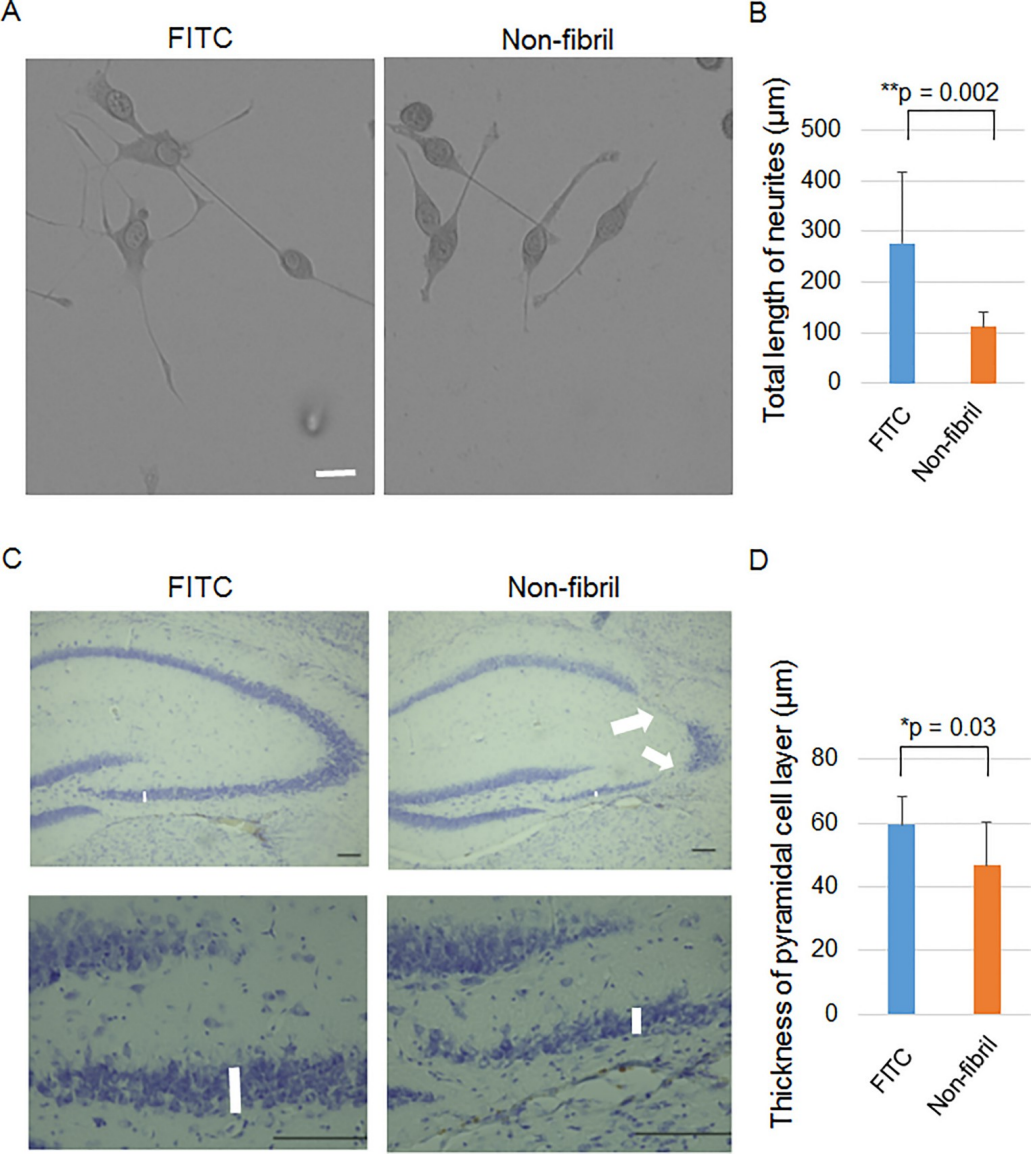
Non-fibril form induces morphological changes in neurons. (A, B) Disturbed differentiation of neuron-like PC12 cells by the non-fibril form. Representative images (A) and quantification of the total length of neurites (B) of PC12 cells in the presence of FITC and the non-fibril form after NGF-induced differentiation. The numbers of biological replicates were 6 for all groups and 2–5 images were taken from each biological replicate. n = 67 and 60 cells for FITC and the non-fibril form, respectively. The average for all cells per biological replicate was used for the Mann-Whitney U test. (C, D) Neuron loss in the hippocampus by the non-fibril form. Representative images are shown in (C). Quantification of the thickness of the pyramidal cell layer (vertical bars in the pictures) in the CA3c region of the hippocampus in Nissl-stained coronal brain sections of mice (D). FITC and the non-fibril form were injected into the hippocampus a month prior. Arrows indicate the regions with extensive neuronal loss. n = 8 and 12 for FITC and the non-fibril form, respectively. ANOVA was applied. Error bars represent SD. *p < 0.05; **p < 0.01. Scale bars, 20 μm (A) and 100 μm (C).

Given broad behavioral changes seen in mice after injection with non-fibril form, we examined the morphology of neuron-like cultured cells and neurons in mouse brains after application of non-fibril form. We used the neurite outgrowth capacity of neuron-like PC12 cells because it was impaired after adding polyalanine (PA) aggregates [16]. NGF-induced differentiation started one day after adding the non-fibril form. The cells were then cultured for 5 days. Quantification of the total length of neurites of PC12 cells revealed that the length was significantly shorter in the presence of non-fibril form than in the presence of FITC (Fig 6A and 6B). This result indicates the deficit in neurite outgrowth capacity induced by the non-fibril form.

One month after the injection of FITC or the non-fibril form in the hippocampus, hippocampal neurons were examined histologically. Extensive loss of pyramidal cells was occasionally observed after injecting the non-fibril form (Fig 6C). For quantification, sets of sections with the same rostral-caudal levels between the two groups were prepared. The thickness of the pyramidal cell layer in the CA3c region, close to the lateral end of the dentate granule cells, was measured (Fig 6C). The non-fibril form induced a significantly thinner pyramidal cell layer than FITC (Fig 6D). Taken together, the non-fibril form of hIAPP 8–20 led to morphological abnormalities in cultured cells and in the brain.

## Discussion

In the present study, we showed that the non-fibril form but not the fibril of hIAPP 8–20 induces changes in spontaneous motor activity and preference for location in the open field in mice. These findings indicate that fibril formation of hIAPP protects brains from functional impairments by hIAPP. hIAPP deposition is found in the hippocampus in Alzheimer disease patients [11]. Our results suggest that the non-fibril form of the hIAPP deposition likely impairs the functions of the hippocampus.

Newly synthesized proteins fold into specific three-dimensional structures to exert their physiological functions [24]. Misfolding of proteins sometimes leads to the formation of toxic aggregate species [24], which disables their physiological functions. Neurodegeneration-causative proteins are the best-studied misfolded proteins. Neurodegenerative polyglutamine (polyQ) diseases [25–30] are caused by expanded CAG repeats in certain genes. Mutant genes are translated into proteins with expanded glutamine repeats, which tend to form aggregates. The aggregation of polyQ-containing proteins is closely related to toxicity because the propensity for aggregation was suggested to be an accurate surrogate for polyQ toxicity rather than the repeat length of polyQ [31]. Endogenous aggregated proteins exert toxicity to cells via multiple mechanisms, such as impaired autophagy, impaired axonal transport, and mitochondrial dysfunction [32]. In addition, aggregated proteins can be transferred between cells [33, 34]. Therefore, endogenous and exogenous aggregates can adversely affect cells. Indeed, we have previously shown that aggregated exogenously applied peptides with long polyQ repeats spontaneously enter neuron-like cells [35]. Functionally, the long polyQ repeat induced retraction of neurites, while the short polyQ repeat had much less retraction [35].

The aggregation dependency of neuronal impairment was studied using exogenous PA with a 13 alanine repeat [16]. Exogenous PA can be spontaneously taken up by cells with high efficiency. The PA aggregates with a higher percentage of β sheet after incubation for 7 days at 37°C, but not those with a lower percentage of β sheet after incubation for 4 h in room temperature, disturbed the behavior of mice [16]. Although the aggregation of exogenous PA with a homopolymeric repeat led to behavioral changes in mice, it remained unclear whether this was also the case in aggregates without homopolymeric repeats. We addressed this issue using hIAPP.

hIAPP-soluble oligomers are the major species that cause toxicity in pancreatic β cells. This concept has been well proven by concurrent time-resolved structural and biological data using hIAPP [12]. hIAPP was incubated up to around 60h at 25°C, and the collected aliquots at several time points were subjected to TEM analysis, the Thioflavin-T assay and β cell viability assay. The hIAPP in the aliquot collected at an incubation time of approximately 60h exhibited a fibril-like appearance and showed high Thioflavin-T fluorescence. When the aliquot was applied to INS-1 β cells, the viability of the cells was not affected (similar to that of non-amyloidogenic rat IAPP) [12]. However, the majority of the hIAPP in aliquots at 0h and around 10h did not show a fibril-like appearance, and application of the two aliquots resulted in low β cell viability with levels of approximately 70% (0h) and 20% (60h) relative to that at 60h [12].

Similar results were obtained using neuron-like cells in this study. hIAPP 8–20 after incubation for a couple of minutes inhibited neurite elongation. Consistent with this observation, a previous report suggested that human recombinant IAPP without pre-incubation decreased the levels of microtubule-associated protein 2, a specific marker for neurite outgrowth, and synapsin, a marker for synaptogenesis, in mouse hippocampal neuronal cells [36]. The non-fibril form but not the fibril form of hIAPP 8–20 located outside the cells likely causes cellular dysfunction after attaching to the cell surface. Consistently, the absorption of IAPP is induced by phosphatidylcholine vesicles with a lipid bilayer [37]. On the cell membrane, soluble oligomers of hIAPP may form membrane channels or be converted into fibrils [38] and the elongation of IAPP fibrils on the membrane could lead to membrane disruption [39, 40]. In addition, preamyloid IAPP intermediates were found to mediate pancreatic β cell proteotoxicity by binding to a receptor for advanced glycation end-products on the cellular surface [41].

One day after injection into the hippocampus, preference for location in the open field was changed in the non-fibril-injected mice. The change, however, was not seen 1 month later. Thus, the behavioral change by the non-fibril form might be due to transient functional changes in the hippocampus. The transient changes in mouse behavior after injuries were often observed. For instance, asymmetrical motor function was recognized 7 days post controlled cortical impact. Then, the alteration was not seen 7 days later (14 days post cortical impact) [42]. Notably, the asymmetrical motor function was not detected 7 days after controlled cortical impact in a microglia-specific inducible Nogo knockout mouse [42]. The knockout mice exhibited a decrease in microglial and astrocyte immunoreactivity and an increase in microglial morphological complexity compared with injury-matched controls [42]. Based on the observations, the authors suggested that the level of tissue inflammation was decreased in the knockout mice after the injury [42]. Similarly, upregulation of multiple inflammatory cytokines was observed within 1 day in rodents given spinal cord injury [43]. In our study, overall levels of tissue inflammation might be increased by the non-fibril form of hIAPP 8–20 in 1 day, which might lead to functional impairments of hippocampal neurons. Alternatively, the frequency of calcium transients which reflects increased neuronal excitability might be changed, as seen in Aβ-treated neurons [44].

In a previous study, an open-field test was performed shortly after IAPP injection, as in the present study. Administration of IAPP into the lateral ventricle of rats inhibited locomotion in an open field at 3 and 6h [40]. The rats did not show behavioral changes 24 hours later [40]. The opposite effects of exogenous IAPP on locomotion between the current study and the previous report might be due to differences in animal species or injection sites. In addition, because the structure of the fibril of hIAPP 8–20 is different from that of full-length hIAPP, the effects of the fibrils of the two are likely different. Contrary to our results using exogenous hIAPP 8–20, movement time in an open field was not increased by the accumulation of IAPP in the hippocampi of hIAPP transgenic mice after a high-fat diet [45]. In mice, Aβ42 deposition has also been observed in the hippocampus, which might hide increased moving time.

Remarkably, defective social behavior by the non-fibril was seen 1 month but not 1 day after the injection. Loss of neurons in the hippocampus found in these mice at 1 month might be responsible for the behavioral change. In contrast, the non-fibril did not change anxiety-like behavior at 1 month. Anxiety is mediated by specific neurotransmitters and/or receptor subtypes in the hippocampus [23]. The non-fibril form of hIAPP 8–20 may not alter the responsible transmitter and receptor systems in the hippocampus.

We administered hIAPP 8–20 into the hippocampus. In contrast, in a previous study, injection of IAPP into the lateral ventricle in rats affected passive and active avoidance behaviors [40]. Therefore, exogenous hIAPP 8–20 in brain regions other than the hippocampus may lead to changes in different behavioral tests. Future studies are needed to clarify the point.

## Abbreviations

Aβ: amyloid beta
IAPP: islet amyloid polypeptide
PA: polyalanine
polyQ: polyglutamine
